# Neonatal point-of-care ultrasound—guidelines for training, credentialing and quality assurance

**DOI:** 10.1038/s41372-025-02367-1

**Published:** 2025-08-01

**Authors:** María V. Fraga, Shazia Bhombal, Courtney Juliano, Minso Kim, Alan M. Groves, Belinda Chan, Yogen Singh

**Affiliations:** 1 Department of Pediatrics, Children’s Hospital of Philadelphia and Perelman School of Medicine, University of Pennsylvania, Philadelphia, PA USA; 2 Department of Pediatrics, Children’s Healthcare of Atlanta/Emory University, Atlanta, GA USA; 3 Department of Pediatrics, Icahn School of Medicine at Mount Sinai, New York, NY USA; 4 Department of Pediatrics, University of California San Francisco, San Francisco, CA USA; 5 Department of Pediatrics, Dell Medical School, University of Texas at Austin, Austin, TX USA; 6 Department of Pediatrics, Division of Neonatology, University of California, UC Davis Children’s Hospital, Sacramento, CA USA; 7 Children’s Healthcare of Atlanta/Emory University, Atlanta, GA USA; 8 Division of Neonatology, University of California, UC Davis Children’s Hospital, Sacramento, CA USA; 9 Division of Neonatology, Ann & Robert H Lurie Children’s Hospital and Northwestern University Feinberg School of Medicine, Chicago, IL USA; 10 Nemours Children’s Health, Cleveland Clinic Tradition Hospital, Port St. Lucie, FL USA; 11 Children’s Hospital of Philadelphia and Perelman School of Medicine, University of Pennsylvania, Philadelphia, PA USA; 12 Dell Medical School, University of Texas at Austin, Austin, TX USA; 13 University of Colorado School of Medicine, Denver, CO USA; 14 University of North Carolina, Chapel Hill, NC USA; 15 Baylor College of Medicine, Texas Texas Children’s Hospital, Houston, TX USA; 16 Children’s Hospital of Los Angeles, Keck School of Medicine, University of Southern Carolina, Los Angeles, CA USA; 17 Icahn School of Medicine at Mount Sinai, New York, NY USA; 18 University of California San Francisco, San Francisco, CA USA; 19 Orlando Health Hospital, Orlando, FL USA; 20 New York-Presbyterian Morgan Stanley Children’s Hospital, New York, NY USA; 21 University of Wisconsin School of Medicine and Public Health and UnityPoint Health Meriter, Madison, WI USA; 22 University of California Irvine School of Medicine, Irvine, CA USA

**Keywords:** Paediatrics, Medical imaging

## Abstract

Point-of-care ultrasound (POCUS) has become essential for diagnosing and managing critically ill newborns. This technology offers rapid, non-invasive assessments and supports bedside clinical decision-making. Although POCUS applications in neonatology continue to expand, there remains a lack of standardized training, certification, and credentialing processes. This paper provides expert-based perspectives and guidelines for implementing neonatal POCUS, focusing on the core components of competency, credentialing, and quality assurance (QA). Recommendations include performing a minimum number of scans for various neonatal applications, integrating competency assessments into training programs, and ensuring a robust image repository and reporting pathway. Neonatal POCUS improves patient care, and establishing clear standards and frameworks will enhance provider performance, and ensure patient safety in neonatal intensive care units (NICUs).

## Introduction

Point-of-care ultrasound (POCUS) in neonatal care has expanded considerably in recent years and has become an essential clinical tool for managing critically ill newborns. Procedural POCUS is increasingly utilized as an adjunct technology to enhance performance by providing real-time visualization of anatomy [1, 2]. Diagnostic POCUS provides valuable information that complements physical examination findings and existing clinical data to support clinical decision-making [3, 4].

Growing evidence has prompted the American Academy of Pediatrics (AAP) to advocate for the use of POCUS in pediatric diagnostic and procedural practices within emergency medicine [5]. Additionally, the European Society for Pediatric and Neonatal Intensive Care (ESPNIC) issued international, evidence-based guidelines for the use of POCUS in pediatric and neonatal intensive care units (NICUs) [6]. More recently, the AAP endorsed a clinical and a technical report supporting the use of POCUS in the NICU for both diagnostic and procedural purposes [7].

While the use of POCUS continues to increase in neonatology, there are currently no standardized training curricula, formal accreditation, or national certification processes exist to guide program development. As the incorporation of POCUS into neonatal care expands, establishing a robust framework for its credentialing and quality assurance (QA) is essential to ensure safe and effective use. This manuscript provides practical guidelines based on expert opinions for implementing neonatal POCUS, focusing on the key components of training, credentialing, and ongoing competency assessment. Establishing clear standards and QA protocols will enhance the utility of this technology and improve patient care.

## Methodology for consensus development and recommendations

The development of consensus and recommendations for the use of POCUS in neonatal care followed a structured and systematic approach to gathering expert opinions and evidence-based insights. Three lead authors (MVF, SB, YS) identified additional expert colleagues who significantly contributed to publications on neonatal POCUS and/or had developed POCUS training courses in the last 10 years. The expert panel selection (co-authors) was conducted prior to the literature review, ensuring representation from a broad range of POCUS fields and incorporation of perspectives that reflect regional variations and clinical practices relevant across the United States. All experts were members of the National Neonatal POCUS Collaborative (NNPC). This approach was intended to capture a comprehensive perspective on the use of POCUS in neonatal care, while also accounting for regional variations and clinical practices prevalent across the United States. A comprehensive review of literature was performed to gather the most up-to-date evidence, focusing on studies from the last ten years, clinical guidelines, and expert opinions regarding the use of POCUS in neonatology. The development of these practice guidelines and recommendations for neonatal POCUS practice involved multiple meetings and feedback exchanges with the expert panel and the NNPC Guidelines Subcommittee members. The guidelines were organized into five subsections, corresponding to key areas of application (heart, lungs, brain, abdomen, and procedural). Within each section, both basic and advanced POCUS applications were defined. Facilitated group discussions addressed specific issues such as training needs, accreditation standards, and competency assessments in neonatal POCUS. The grade of recommendation set forth by the Oxford Center for Evidence-Based Medicine (CEBM) was used for the level of evidence: A-level = Consistent level 1 studies, B-level = Consistent level 2 or 3 studies or extrapolations from level 1 studies, C-level = Level 4 studies or extrapolations from level 2 or 3 studies, and D-level = Level 5 evidence or troubling inconsistent or inconclusive studies at any level. The draft recommendations were shared with a broader group of neonatal POCUS colleagues for feedback through the NNPC. This ensured that the final recommendations were practical, feasible, and aligned with clinical practice. Feedback from these stakeholders was incorporated into the final document. The final set of recommendations was prepared in accordance with the international Appraisal of Guidelines, Research and Evaluation (AGREE) [8] and was reviewed and approved by the expert panel, ensuring that they reflected the committee’s consensus. The expert consensus was developed using majority vote among the panelists, following multiple online discussions. The resulting recommendations offer an  evidence-based framework for implementing neonatal POCUS, with an emphasis on safe practice, QA, and ongoing professional development.

## Scope of practice in neonatology: basic and advanced applications

With the expanding use of POCUS in the NICU, new clinical applications continue to emerge. As of 2025, position statements from the AAP [7], the American Society of Echocardiography (ASE) [9], and ESPNIC [6] offer valuable guidance and frameworks for POCUS use in the NICU.

A comprehensive list of neonatal POCUS applications was developed following a literature review [10–47] with each application assigned a corresponding  level of evidence. All diagnostic and procedural applications were categorized as either basic or advanced (Tables 1A and 1B). The list of basic POCUS applications is intended to serve as a foundational framework for developing a core neonatal POCUS curriculum. These basic indications are commonly used in the NICU and can typically  be mastered by learners during the course of NICU fellowship training. In contrast, advanced indications require more specialized ultrasound training to achieve proficiency.

**Table 1:** A Basic neonatal POCUS applications. B Advanced neonatal POCUS applications.

System	Indication	Summary statement	Level of Evidence
**A**			
**Diagnostic Abdomen**	Bladder Assessment	POCUS *is helpful* to assess bladder volume in neonates with oliguria/anuria.	B
	Ascites	POCUS *is helpful* to assess for intraperitoneal free fluid in neonates	B
**Diagnostic Cardiac**	Qualitative Assessment of Cardiac Function	POCUS *may be* used to assess cardiac contractility and function in neonates with clinical decompensation	C
	Cardiac Filling and Fluid Responsiveness	POCUS *may be* used to assess fluid status/cardiac filling and response to volume resuscitation in neonates with clinical decompensation	C
	Pericardial Effusion	POCUS *is helpful* to assess for pericardial effusion in neonates with clinical decompensation	B
	Heart-rate Assessment	POCUS *may be* used to assess heart rate during neonatal resuscitation	C
**Diagnostic Central Line Assessment**	Umbilical Venous Catheter Position Confirmation	POCUS *is recommended* to assess umbilical venous catheter tip position in neonates	A
	Umbilical Arterial Catheter Position Confirmation	POCUS *may* be used to assess umbilical arterial catheter tip position in neonates	C
	Peripherally Inserted Central Catheter (PICC) Position Confirmation	POCUS *is recommended* to assess PICC line tip position in neonates	A
**Diagnostic Cranial**	Detection of Severe (Grade III/IV) Intraventricular Hemorrhage	POCUS *is helpful* to assess for severe IVH in preterm neonates with clinical decompensation	B
**Diagnostic Pulmonary**	Pneumothorax	POCUS *is recommended* to assess for pneumothorax in neonates	A
	Pleural Effusion	POCUS *is helpful* to assess for pleural effusion in neonates	B
**Procedural**	Peripheral Vessel Cannulation (Venous or Arterial)	POCUS *is recommended* to guide peripheral vessel cannulation in neonates	A
	Lumbar Puncture Assessment for Site Identification	POCUS *is recommended* to assess landmarks and identify appropriate site for performing lumbar puncture in neonates	A
	Chest-tube Insertion/Thoracentesis	POCUS *is helpful* to guide chest tube insertion and thoracentesis in neonates	B
	ETT Position (Intratracheal)	POCUS *is helpful* to confirm ETT position as intratracheal in neonates	B
	Suprapubic Tap	POCUS *is recommended* to guide catheter insertion during suprapubic tap in neonates	A
	Paracentesis	POCUS *is helpful* to guide paracentesis in neonates	B
	Pericardiocentesis	POCUS *is helpful* to guide percardiocentesis in neonates	B
**B**			
**Diagnostic Abdomen**	Characterization of ascites	POCUS is helpful to assess the nature of ascites (transudative vs exudative, simple vs complex) in neonates	B
	Bowel Peristalsis	POCUS *may be* used to assess bowel peristalsis in neonates	C
	Bowel vascularity	POCUS *may be* used to assess bowel vascularity in neonates	C
	Evaluation of Necrotizing Enterocolitis (NEC)	Evaluation of Necrotizing Enterocolitis (NEC)	A
**Diagnostic Central Line Assessment**	ECMO Cannula Position Confirmation	POCUS *may be* used to assess ECMO cannula tip position in neonates	C
**Diagnostic Pulmonary**	Pulmonary Edema	POCUS *may be* used to diagnose pulmonary edema and to assess response to therapy in neonates	C
	Atelectasis	POCUS *may be* used to diagnose lung atelectasis and to assess response to recruitment maneuvers in neonates	C
	Transient Tachypnea of Newborn (TTN)	POCUS *is recommended for use* in diagnosing TTN in neonates	A
	Respiratory Distress Syndrome (RDS)/Surfactant Deficiency	POCUS *is recommended* for use in diagnosing RDS and in assessing the need for surfactant replacement therapy in neonates	A
	Meconium Aspiration Syndrome (MAS)	POCUS *may be* used to diagnose meconium aspiration in neonates	C
	Pneumonia	POCUS *is helpful* for use in diagnosing pneumonia in neonates	B
	Bronchopulmonary Dysplasia (BPD)/Lung Severity Score	POCUS *is helpful* to assess the severity of lung disease and the likelihood of progression to BPD in neonates	B
**Procedural**	Umbilical Venous Catheter Dynamic Insertion	POCUS *may be* used to guide umbilical venous catheter insertion in neonates	C
	Femoral Catheter Insertion	POCUS *is recommended* for use during femoral catheter insertion in neonates	A
	Internal Jugular Catheter Insertion	POCUS *is recommended* for use during internal jugular catheter insertion in neonates	A
	Subclavian Venous Catheter Insertion	POCUS *is helpful* for use during subclavian catheter insertion in neonates	B
	ETT Positioning (Depth)	POCUS *is helpful* to confirm ETT position in neonates	B

## Competency, credentialing, and institutional privileges

Demonstrating competency, meeting credentialing requirements, and obtaining privileges in neonatal POCUS are essential to ensure that practitioners possess the necessary skills and knowledge to use ultrasound both effectively and safely. Competency in POCUS pertains to the individual provider and encompasses several key elements: understanding the clinical indications for its use, acquiring the technical skills to obtain high-quality images, accurately interpreting those images, and integrating the findings into clinical decision-making. Credentialing refers to institution specific criteria to define the scope of practice and its integration into clinical workflows. This process is critical for enabling  practitioners to base medical decisions on POCUS findings, include images and interpretations in the medical record, and submit billing claims for POCUS using the appropriate Current Procedural Terminology (CPT) codes [48]. POCUS privileges are granted by the hospital and formally authorize a provider to perform POCUS within the institution.

**Fig. 1:**
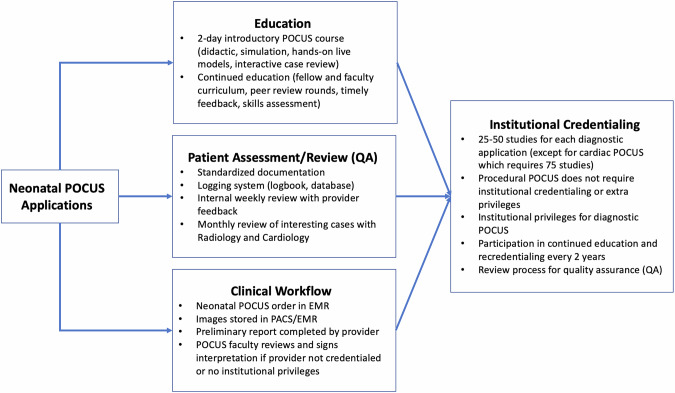
Proposed framework for neonatal POCUS program development. EMR electronic medical record, QA quality assurance, PACS picture archiving and communication system.

Achieving competency requires both theoretical knowledge and hands-on experience, including demonstrated proficiency in performing and interpreting ultrasound exams. Performing a set number of POCUS examinations is a component of reaching competency, which can be further assessed by POCUS experts. However, standardized training guidelines specific to the NICU clinical scope are currently lacking. The Emergency Medicine and the Pediatric Emergency Medicine Expert Guidelines recommend performing 25 to 50 POCUS examinations for each organ system to ensure proficiency [49, 50]. Extrapolating from these guidelines, the neonatology community recommends performing 25-50 studies per each diagnostic application with a mix of normal and abnormal exams, excluding cardiac POCUS. Currently, there is insufficient evidence to justify a specific minimum number of scans required to determine competency in neonatal cardiac POCUS training. However, due to the numerous cardiac views and multiple applications of neonatal cardiac imaging, it is recommended that trainees perform at least 75 scans, including a minimum of 25 exams focused on line placement and heart function evaluation and 50 exams focused on global systolic cardiac function, pericardial effusion and volume status assessment [9]. These numbers should be viewed as expert recommendations rather than strict requirements. Furthermore, imposing fixed minimum scan numbers may unnecessarily delay the implementation of proven POCUS techniques from reaching the bedside, particularly in simpler applications or when practitioners already have significant scanning experience in other organ systems. Therefore, a more pragmatic approach of competency-based rather than time-based assessment of skills should be considered. This competency-based approach may include synchronous evaluation methods such as observation at the patient bedside or via simulation and may also include asynchronous practices such as interactive image review for evaluation of acquisition quality and interpretation accuracy. These educational practices can also be incorporated into a QA program to ensure ongoing safe and effective practice. Figure 1 depicts a proposed framework for neonatal POCUS program development including training, a QA process and clinical workflow leading to institutional credentialing.

While the use of ultrasound for procedural guidance (e.g., central line placement, thoracentesis, paracentesis) is typically considered an adjunct technology that enhances provider performance and patient safety, it does not require formal credentialing or institutional privileging beyond baseline procedural credentialing. However, competency, training, and adherence to safety standards remain critical components of its use in clinical practice.

Collaborating with existing multidisciplinary POCUS teams within an institution can help leverage available resources and support the inclusion of neonatal POCUS within the hospital-wide credentialing framework.

## Quality assurance process

QA in neonatal POCUS is essential to ensuring the safety of patients and healthcare institutions. This process involves systematic measures to verify the accuracy, reliability, and safety of ultrasound examinations. An inadequate QA process can compromise patient care and increase the risk of legal liability. Several organizations, including the ACEP (American College of Emergency Physicians) and ASE, have issued policy statements and guidelines related to POCUS QA [49–52]. To maintain patient safety, ongoing education of POCUS practitioners is vital. Practitioners should receive specialized training, demonstrate competency, and acquire appropriate credentialing and privileges. Regular competency assessments and continuing education help ensure that practitioners remain up-to-date with current guidelines and best practices. Additionally, practitioners should work within a clearly defined scope of POCUS practices to uphold safety standards. All POCUS images should be archived along with documentation of findings and interpretations to facilitate regular audits. The quality of these images should be reviewed to ensure they meet established standards. Similarly, findings and interpretations should undergo audits for accuracy, with interpretations validated against patient outcomes, results from other diagnostic tests, and/or surgical or pathology evaluations. It is essential that POCUS practitioners receive timely, constructive feedback in a nonjudgmental manner to support continuous improvement and ensure high-quality care. Institutions play a vital role in QA by establishing a review committee and supporting a dedicated POCUS program director. Advanced information technology may have electronic medical records linking POCUS order, POCUS machine image acquisition, image transmission to the patient chart, and documentation of image interpretation.

Initiating a POCUS program can present several challenges, particularly in establishing a robust QA process. Common obstacles include a shortage of POCUS experts within the subspecialty, the lack of standardized competency and educational frameworks, and resistance from administrators to provide necessary support [53]. During the early stages of program development, it is important to build relationships with multidisciplinary POCUS providers within the institution to support various aspects of programmatic development, including expertise in image acquisition and interpretation. Until a dedicated internal QA team is established, auditing all images and collaborating with radiologists, cardiologists, and local POCUS experts for quality control is imperative. In conclusion, the QA process for neonatal POCUS is a comprehensive, ongoing effort that encompasses education, equipment maintenance, institutional policy development and continuous evaluation. This multifaceted approach ensures that neonatal POCUS is performed to the highest standards, upholding both patient safety and the integrity of the healthcare system.

## International relevance and adaptability of POCUS guidelines

International evidence-based ESPNIC POCUS guidelines provide comprehensive recommendations for the use of POCUS in the neonatal and pediatric intensive care units [5]. However, these are joint guidelines intended for use in both neonates and older children. In contrast, the currently proposed guidelines are neonatal specific and provide guidance on the basic and advanced applications. The authors believe these newly proposed guidelines can be adapted easily for international use in any neonatal settings and they are complimentary to the ESPNIC POCUS guidelines.

## Conclusions

In summary, the use of POCUS in neonatology has significantly advanced, offering benefits in diagnostic and procedural guidance. However, to ensure its safe and effective use, it is essential to establish structured frameworks for competency, credentialing, and QA process. These frameworks should include training programs, ongoing competency assessments, and timely feedback for practitioners, as well as QA processes to monitor image quality and clinical accuracy. As neonatal POCUS evolves, institutions should focus on supporting these efforts through adequate resources and infrastructure.
